# A randomized, open-label clinical trial in mild cognitive impairment with EGb 761 examining blood markers of inflammation and oxidative stress

**DOI:** 10.1038/s41598-023-32515-6

**Published:** 2023-04-03

**Authors:** Xavier Morató, Marta Marquié, Juan Pablo Tartari, Asunción Lafuente, Carla Abdelnour, Montserrat Alegret, Sara Jofresa, Mar Buendía, Ana Pancho, Núria Aguilera, Marta Ibarria, Susana Diego, Rosario Cuevas, Laia Cañada, Anna Calvet, Ester Esteban-De Antonio, Alba Pérez-Cordón, Ángela Sanabria, Itziar de Rojas, Raúl Nuñez-Llaves, Amanda Cano, Adelina Orellana, Laura Montrreal, Pilar Cañabate, Maitée Rosende-Roca, Liliana Vargas, Urszula Bojaryn, Mario Ricciardi, Diana M. Ariton, Ana Espinosa, Gemma Ortega, Nathalia Muñoz, Núria Lleonart, Emilio Alarcón-Martín, Mariola Moreno, Silvia Preckler, Natalia Tantinya, Maribel Ramis, Ana Belen Nogales, Susanna Seguer, Elvira Martín, Vanesa Pytel, Sergi Valero, Miren Gurruchaga, Lluís Tárraga, Agustín Ruiz, Mercè Boada

**Affiliations:** 1 Ace Alzheimer Center Barcelona-Universitat Internacional de Catalunya, Barcelona, Spain; 2 Networking Research Center on Neurodegenerative Diseases (CIBERNED), Instituto de Salud Carlos III, Madrid, Spain; 3 Department of Neurology and Neurological Sciences, Stanford University School of Medicine, Stanford, CA USA

**Keywords:** Cognitive ageing, Molecular neuroscience, Biomarkers, Medical research, Dementia

## Abstract

Although beta-amyloid (Aβ) and phosphorylated tau remain the preferred targets for disease-modifying treatments (DMT) against Alzheimer’s disease (AD), part of the pathophysiological mechanisms of cognitive impairment are related to neuroinflammation and oxidative stress. In mild cognitive impairment (MCI), a prodromal stage of AD and other neurodegenerative conditions, the joint appearance of inflammation, oxidative stress, and metabolic alterations are the common pathways of neurotoxicity and neurodegeneration. The standardized extract of *Ginkgo biloba* EGb 761 interferes with the pathogenic mechanisms involved in both the development of cognitive impairment due to AD and that of vascular origin. The primary objective of this study is to compare changes in the levels of blood markers of inflammation and oxidative stress after treatment with EGb 761 in a cohort of 100 patients with MCI. In addition, we aim to assess changes in these blood markers during an additional 12-month extension phase in which patients in the control group will also receive EGb 761 and patients in the active group will extend their treatment duration. Secondary objectives include comparing changes in neuropsychiatric and cognitive test scores between the baseline (v0) and 12-month visits (v2). This study is a Phase IV, single-center, randomized, open-label, parallel-group clinical trial consisting of the 12-month follow-up of a cohort of participants with MCI [Global Deterioration Scale (GDS) = 3] and an extension with an additional 12-month follow-up. During the first 12 months, participants will be randomized into two arms: in one arm, patients will receive 1 daily tablet of EGb 761 240 mg orally (study group, *n* = 50), while in the other arm, patients will not receive EGb 761 and will undergo the same assessments as the treated group (control group, *n* = 50). After the first 12 months of the study, patients in the EGb 761-treated group will continue treatment, and patients in the control group will be offered one EGb 761 240 mg tablet per day orally. All participants will be monitored for an additional 12 months. A battery of blood markers of inflammation and oxidative stress will be quantified at v0, v1, v2, v3, and v4. The Olink Proteomics panel of inflammation markers (https://www.olink.com/products/inflammation/) will be used to evaluate 92 proteins associated with inflammatory diseases and related biological processes. The second panel measures 92 proteins involved in neurological processes. At v0, v2, and v4, neuropsychological and neurological evaluations will be conducted in addition to vital signs and anthropometric studies using a body composition monitor with bioimpedance technology (Tanita). Sixty percent of the 100 MCI patients recruited were women. The mean age was 73.1 years, and the mean time between symptom onset and MCI diagnosis was 2.9 years. The mean Mini-Mental State Examination (MMSE) score was 26.7. Depressive and anxiety disorders, as well as vascular risk factors, were the most frequent comorbidities among the cohort. The study is still ongoing, and results for the first year of treatment (v0, v1, v2) are expected by 2023. Individuals with MCI have an elevated risk of developing dementia. EGb 761 is used worldwide for the symptomatic treatment of cognitive disorders due to its neuroprotective effects. In experimental models and clinical observational studies, EGb 761 has shown strong antioxidant and anti-inflammatory activity. As a result, this study has been proposed to evaluate the antioxidant and anti-inflammatory effects on plasma markers and their potential clinical correlation with the progression of cognitive decline in patients with MCI.

**Trial registration:** Registro Español de estudios clínicos (REec) number 2020-003776-41, ClinicalTrials.gov Identifier: NCT05594355.

## Introduction

Mild cognitive impairment (MCI) is an impairment in one or more cognitive domains that is higher than expected for an individual’s age and level of education. However, it is not associated with functional deficits or severe enough to establish a diagnosis of dementia^1^. MCI prevalence increases with age, ranging from 6.7% between the ages of 60 and 64–25.2% between the ages of 80 and 84^2^.

Although the underlying pathophysiology of MCI is multifactorial, this clinical condition is considered a prodromal stage of dementia, including Alzheimer’s disease (AD)^3^. After a 2-year follow-up, cumulative dementia incidence among individuals older than 65 with MCI is approximately 15%^2^. Importantly, the risk of progression to dementia is influenced by the various clinical MCI endophenotypes (amnestic vs. non-amnestic^1^, probable vs. possible^4^, and the presence of abnormal AD core biomarkers)^5^. A 60-month longitudinal follow-up study of 550 MCI patients assessed at ACE Alzheimer Center Barcelona found that those with probable amnestic endophenotypes had an 8.5-fold higher risk of progression to dementia (mainly AD) than those with a possible non-amnestic type, who had the best prognosis^6^. Another study from our group showed that the probable-amnestic MCI subtype correlates best with AD biomarkers, as shown by a lower hippocampal volume on MRI, lower glucose uptake on ^18^F-fluorodeoxyglucose-positron emission tomography (PET-FDG), and higher amyloid uptake on Pittsburgh compound B PET (PET-PiB)^7^. Early detection of cognitive deficits in the MCI stage is crucial for better understanding the molecular mechanisms involved in the pathogenesis of neurodegenerative disorders, developing potential therapeutic targets, and offering dementia prevention strategies.

A series of brain changes associated with aging leads to a decrease in the processes of adaptation and response, which can result in cognitive impairment and/or dementia. Although the causes of these changes vary, inflammation and oxidative stress explain a portion of the pathophysiological mechanisms underlying these abnormalities in brain functions^8^. Chronic neuroinflammation is now thought to play a central role in AD pathology, and recent studies have identified several inflammation pathway genes associated with the risk of AD^9^. Recent publications emphasize the importance of inflammatory and oxidative processes that impact neuronal mitochondrial function in the pathogenesis of cognitive impairment, both of vascular origin and of AD itself^10–12^. Neuroinflammation causes neuronal damage by releasing inflammatory cytokines and activating microglia through membrane receptors and nuclear activation factors. This neuroinflammatory phenomenon also affects neuronal plasticity, altering the genesis and maintenance of long-term potentiation and causing hippocampus-dependent memory impairment. Oxidative stress and the production of free oxygen radicals also cause toxic effects in aging brains, primarily due to lipid peroxidation and DNA damage^13^. In AD, experimental evidence suggests that the joint appearance of inflammation, oxidative stress, and metabolic alterations is the common initial pathway of neurotoxicity and neurodegeneration. Research in the areas of oxidation and inflammation through markers of these mechanisms in patients with different MCI endophenotypes (amnestic vs. non-amnestic, possible vs. probable) can contribute to a greater understanding of the actual impact of these factors on the progression of these neurodegenerative processes.

EGb 761 is a standardized extract of *Gingko biloba L.* dried leaves with a composition adjusted according to the specifications of the European Pharmacopoeia: 22–27% flavonoid glycosides, 5–7% terpene lactones (2.8–3.4% ginkgolides A, B, and C, and 2.6–3.2% bilobalide), and less than 5 parts per million ginkgolic acids^14^. The quality and composition of EGb 761 are regularly monitored, and the content of the most important ingredients that contribute to efficacy (e.g., terpene lactones, flavone glycosides) and compliance with limits of unwanted compounds (e.g., ginkgolic acids) are evaluated. Additionally, general parameters such as ash, color, and particle size are analyzed (a total of 18 parameters)^15^. EGb 761 interferes with the pathogenic mechanisms involved in both the development of cognitive impairment due to AD and that of vascular origin. Due to its powerful antioxidant effect, which eliminates free radicals, it improves mitochondrial function at the neuronal level and protects membranes from lipid peroxidation^16^. The compound also stimulates neurogenesis in the hippocampus, increases neuroplasticity^17^, and inhibits β-amyloid aggregation and toxicity^18^. The extract decreases blood viscosity and improves cerebral microperfusion^19^. All this information suggest that EGb 761 interferes with different neuropathological pathways associated with AD, vascular dementia, and mixed dementia.

In two randomized, double-blind, placebo-controlled clinical trials in patients with MCI, EGb 761 demonstrated superiority over placebo in terms of cognitive function improvement, with neuropsychiatric symptoms (NPS) showing a safety profile comparable to placebo in terms of the number and severity of adverse events^20,21^. In the setting of mild-moderate dementia, EGb 761 at a dose of 240 mg/day orally has been shown to be superior to placebo in improving cognitive functions, NPS, activities of daily living, quality of life, and clinical global impression, as demonstrated in several recent meta-analyses, including 7 double-blind, placebo-controlled, randomized clinical trials^22,23^. These same meta-analyses confirmed the drug’s good safety profile, presenting a number of adverse events similar to or even lower than those seen in placebo-treated groups.

Regarding changes in neuroinflammatory biomarkers in patients treated with EGb761, a study carried out by Spanish researchers on a population of 65 patients with MCI treated with EGb 761 at a dose of 240 mg/day and followed for 6 months reported a decrease in certain peripheral inflammatory markers (CRP, cystatin, and IL-6) that were elevated at baseline, accompanied by increased relative to baseline levels of antioxidant levels (GSH glutathione, glutathione rate, hemolyzed catalase, catalase protein, and superoxide dismutase)^24^. Due to the potential impact of EGb 761 on the underlying inflammatory and oxidative processes in the evolution of cognitive impairment, we planned a study to investigate these effects and analyze their impact on the clinical progression of MCI. Thus, the main objective of this clinical trial, which consists of a 12-month follow-up of a cohort of 100 participants with MCI and an extension with an additional 12-month follow-up period, will be to assess changes in the levels of blood markers of inflammation and oxidative stress after treatment with EGb 761.

## Methods

### Study design

The ACE-2020-EGb 761 study is a Phase IV, single-center, randomized, open-label, parallel-group clinical trial (CT) performed at the Ace Alzheimer Center Barcelona. The study consists of a cohort of individuals with MCI (GDS = 3) followed up for 12 months. Later, an extension of the study was approved, allowing for an additional follow-up period of 12 months. The protocol version 2 extension was approved on 31/01/2022.

**Figure 1:**
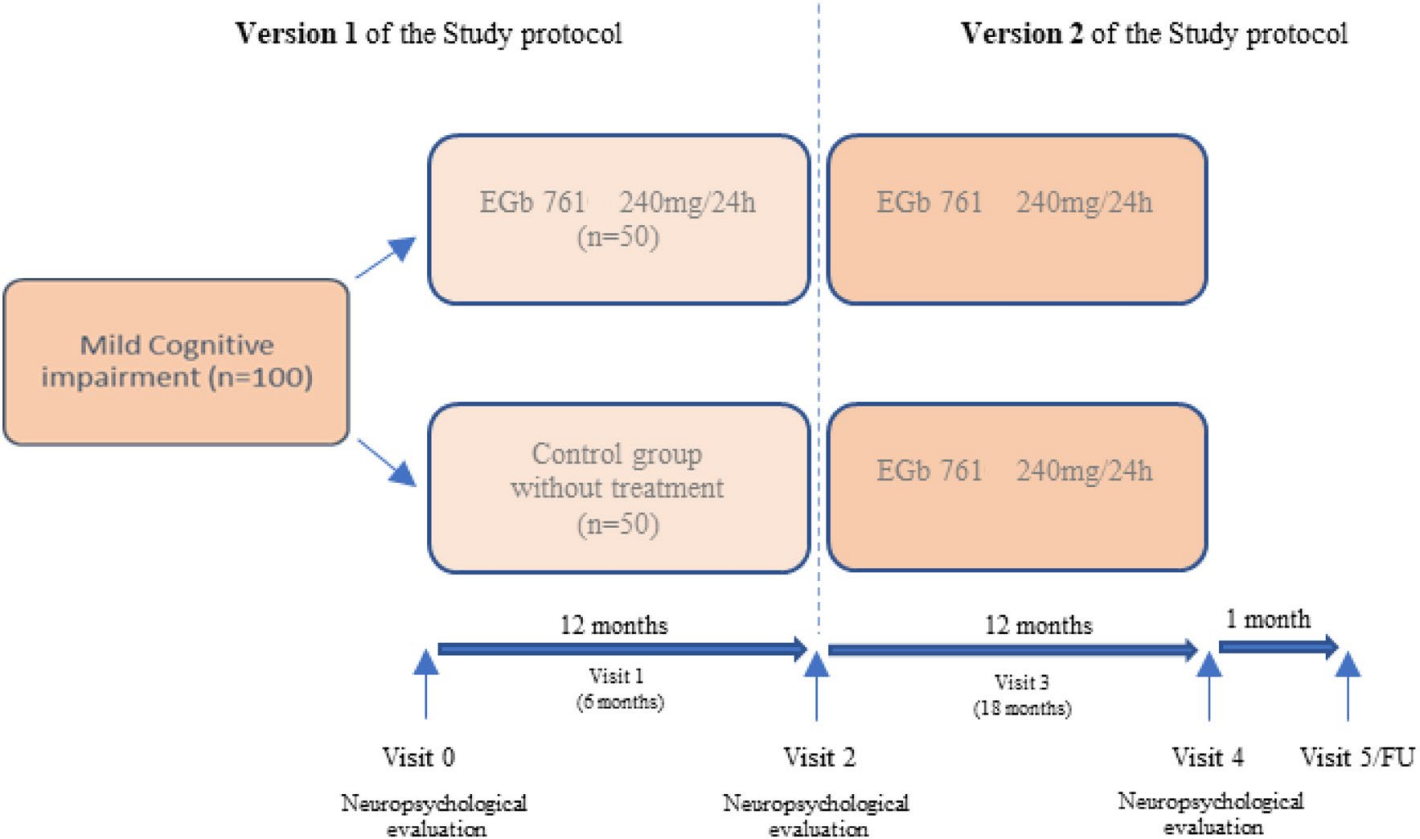
ACE-2020-EGb761 Study Flow Chart. The ACE-2020-EGb761 study was initially designed as a Phase IV, single-center, randomized, open-label, parallel-group clinical trial consisting of the 12-month follow-up of a cohort of participants with MCI (version 1). In January 2022, an extension with an additional 12-month follow-up period was approved.

During the first 12 months of the study, participants were randomly assigned to one of two treatment groups. To achieve allocation concealment, randomization was independently conducted online using a web-based application. The randomization was balanced and non-stratified. In the first arm, MCI patients were treated with 1 daily tablet of EGb 761 240 mg orally (study group, *n* = 50), and in the second arm, MCI patients with identical clinical characteristics were not treated with EGb 761 but underwent the same evaluations as the treated group (control group, *n* = 50) (Fig. 1).

At the end of the first year of follow-up (v2), all participants who completed the study were offered the opportunity to continue in an extended phase consisting of an additional year of follow-up. During this extended period, participants in the control group were offered to begin treatment with 1 daily tablet of EGb 761 240 mg orally for 12 months (Fig. 1). In addition, participants who were initially assigned to the study group continued to take 1 daily tablet of EGb 761 240 mg orally during this extension period, as they did during the initial phase. All participants who wanted to continue participating in the study had to sign a new informed consent approved by the Ethics Committee, which reviewed the protocol amendment to include this extended phase.

The maximum total duration of the study for each participant is 2 years. Participants will have 6 visits: a baseline visit (v0), a follow-up visit at 6 months (v1), a follow-up visit at 12 months (v2), a follow-up visit at 18 months (v3), a follow-up visit at 24 months (v4, final), and a follow-up visit (v5) 1 month after the final visit. The procedures for each visit are completed within a maximum of 30 days.

In the study group receiving 1 daily tablet of EGb 761 240 mg orally, the medication was dispensed at the Ace Alzheimer Center Barcelona’s Pharmacy every 3 months, with control of therapeutic compliance, adverse events, and an accounting of the returned medication using an ad hoc questionnaire to monitor treatment adherence. This questionnaire also includes four items with dichotomous response categories with yes or no to assess the compliance.

### Eligibility criteria

To be included in the study, participants had to meet all the inclusion criteria and none of the exclusion criteria.

The inclusion criteria were the following: age between 50 and 85 years, a diagnosis of mild cognitive impairment according to Petersen criteria^25^, a Global Deterioration Scale (GDS) score of 3^26^, a Clinical Dementia Rating (CDR) score of 0.5^27^, the ability to comply with the study protocol in the opinion of the investigator, and signing an informed consent form.

The exclusion criteria were the following: diagnosis of dementia (GDS = 4–7)^26^, severe visual and/or auditory deficits that could prevent neuropsychological evaluation, severe psychiatric pathology, hemorrhagic diathesis or anticoagulant treatment, active treatment with cholinesterase inhibitors or memantine, history of epilepsy or alcoholism, galactose intolerance, glucose or galactose malabsorption, and previous treatment with EGb 761.

### Objectives

The main objective of the study is to compare changes in blood levels of inflammation and oxidative stress biomarkers between baseline (v0) and follow-up visits at 6 (v1) and 12 months (v2) between the study group (patients with MCI receiving treatment with 1 tablet daily of EGb 761 240 mg orally) and the control group (patients with the same clinical characteristics without treatment with EGb 761).

The following were the secondary goals: (1) comparing neuropsychiatric and cognitive test scores between the study group and the control group at baseline (v0) and 12-month follow-up (v2) visits; (2) obtaining data on blood levels of inflammation and oxidative stress biomarkers after a 24-month follow-up period in both study groups; (3) evaluating changes in cognitive and neuropsychiatric test scores and blood biomarkers of oxidation and inflammation after 24 months of follow-up between the group treated with EGb761 since the beginning of the study and the group treated only with EGb761 during the last 12 months of the study (extension phase); and (4) obtaining safety data over a 2-year treatment period.

### Sample size

The sample size was determined based on the number of participants required to detect significant changes in blood levels of inflammation and oxidation biomarkers between baseline visit (v0) and follow-up visits at 6 (v1) and 12 months (v2) in patients with MCI in the study group (treated with 240 mg of EGb 761 daily) compared to the control group. For this purpose, cystatin (mg/dL) was chosen as the reference variable since it has a known standard deviation. The average values and standard deviation of cystatin were obtained from an observational study in which the levels of cystatin in the blood of patients with MCI treated with EGb 761 at doses of 240 mg daily were determined (Gil P, 2018).

Assuming that the study group’s cystatin level will be 0.97 mg/dL at the end of treatment and that the control group will maintain a cystatin level of 1.40 mg/dL, as well as a common standard deviation of 0.6, an alpha risk of 5%, a statistical power of 95%, and a drop-out rate of 20%, 50 participants per group would be required.

### Statistical analysis

Using descriptive statistics, we present the baseline sociodemographic and clinical characteristics of the sample. Categorical variables are presented as percentages, while continuous variables are presented as means and standard deviations. SPSS version 26.0 software (SPSS Inc., Chicago, IL) was used to analyze baseline differences in demographic, clinical, and cognitive characteristics between the study and control groups. At baseline, differences in demographic, Tanita, cardiovascular, and neuropsychological characteristics between study and control groups were analyzed using a T-test (continuous variables) or Fisher’s exact test (categorical variables). The level of statistical significance was set at p < 0.05.

After the completion of the study, several linear mixed models with repeated measurements will be used to assess the differences included as outcome measures. The group variable (2 categories: study vs. control) and the time factor (2 moments: v0 and v2) will be considered independent model factors. The various blood markers of inflammation will be considered dependent variables. The interaction group x time will be considered the main target of each model executed (for each blood marker). If any relevant clinical or demographic variable (age, gender, educational level, etc.) is found to be unbalanced between the two groups, it will be incorporated into the model as a covariate.

### Main outcome measures

#### Blood biomarkers

Two panels of human protein biomarkers from Olink Proteomics (https://www.olink.com/products-services/target/) will be used to assess blood biomarkers. The first panel includes 92 proteins associated with inflammatory diseases (i.e., VEGF, IL-6, IL-1B, INF-gamma, and TNF), while the second includes 92 proteins involved in neurological and oxidative processes (GDNF, GFD, SOD2, Catalase, and GGT1, among others). Participants’ blood samples were collected in an EDTA BD VACUTAINER SSTII DE. The samples were then centrifuged at 2000*g* for 10 min at 4 °C, and the plasma was aliquoted in SARSTEDT tubes and stored at − 80 °C. Blood samples obtained at baseline (v0), 6 months (v1), 12 months (v2), 18 months (v3), 24 months (v4), and the last follow-up visit (1 month after the last drug administration; v5) will be sent to Olink Proteomics for blood protein quantification (Table 1). Multiple testing corrections methods will be implemented for the proteomic analysis.

**Table 1:** Data collection and procedures per study visit.

Visit	V0	V1	V2	V3	V4	V5 (FU)
Months from the baseline	0	6	12	18	24	25
Procedure						
Informed consent	X		X			
Eligibility	X					
Medical history	X					
Vital signs and weight	X		X		X	
Anthropometric study (Tanita)	X		X		X	
Neurological examination	X		X		X	X
Adverse events		X	X	X	X	X
Concomitant medication	X	X	X	X	X	X
Medication dispensing	X	X	X	X		
Medication adherence questionnaire		X	X	X	X	
Assessment						
Blood drawn and plasma obtention	X	X	X	X	X	X
Mini-Mental State Examination (MMSE)*	X		X		X	
Clinical dementia rating (CDR)	X		X		X	
Hachinski Ischemic Scale	X		X		X	
Neuropsychiatric Inventory Questionnaire (NPI)*	X		X		X	
Global Deterioration Scale (GDS)	X		X		X	
Geriatric Depression Scale	X		X		X	
Blessed Scale	X		X		X	
NBACE**	X		X		X	
HADS***	X		X		X	

*Spanish version; **Fundació ACE Neuropsychological Battery; ***HADS: the Spanish version of Hospital Anxiety and Depression Scale. *FU* follow-up.

### Further outcome measures

#### Clinical and neurological assessment

All participants are clinically evaluated at baseline (v0), 12 (v2), and 24 months (v4). As part of this evaluation, a neurologist or geriatrician conducted a structured anamnesis with the patient and an informant. Data collected included age, sex, educational level, medical history, cardiovascular risk factors, and treatment received. A comprehensive neurological examination was performed, and the following tests were administered: the Spanish version of the Mini-Mental State Examination test^28^, the Hachinski Ischemic Scale^29^, the Global Deterioration Scale^30^, the Geriatric Depression Scale^31^, the Clinical Dementia Rating^32^, and the Blessed Dementia Scale^33^.

Neuropsychiatric symptoms were assessed using the Spanish version of the Neuropsychiatric Inventory Questionnaire (NPI-Q)^34^. The NPI-Q is a simplified clinical scale used to assess dementia-related behavioral disturbances in 12 domains (delusions, hallucinations, agitation/aggression, depression/dysphoria, anxiety, elation/euphoria, apathy/indifference, disinhibition, irritability/lability, aberrant motor behavior, sleep and nighttime behaviors, and appetite and eating disorders)^35^. In our study, the NPI-Q was administered by trained physicians, and patient information was provided by a reliable informant (family member or close relative). A change in the last month was classified as present or absent for each of the 12 domains (dichotomous variable).

Data from historical neurological examinations performed at Ace Alzheimer Center Barcelona’s Memory Clinic within the 3 months prior to the participant’s inclusion were used for the current study.

#### Neuropsychological assessment

A neuropsychological evaluation was performed at the baseline (v0), 12-month (v2), and 24-month (v4) follow-up visits. Neuropsychological assessments were conducted by raters blinded to the treatment. The Fundació ACE (N-BACE)^36^ neuropsychological battery was administered in person or telematically indistinctly, has an approximate duration of 45 min, and consists of the following tests: (1) orientation to reality (temporal, spatial, and personal); (2) attention and verbal working memory: direct digit subtests of the Wechsler Adult Intelligence Scale-III (WAIS-III), subtest inverse digits of the WAIS-III; (3) learning ability and long-term verbal memory: word list (Wechsler Memory Scale-III) (The Psychological Corporation, 1997); (4) language: Boston Naming Test (an abbreviated version of 15 items), repetition of sentences, verbal comprehension of simple, semi-simple, and complex orders, verbal fluency with a slogan (phonetics and semantics); (5) visual gnosis: Poppelreuter’s test, Luria’s test, test of 15 objects; (6) imitation motor and ideomotor praxias, subtest “cubs” of the WAIS-III; (7) prefrontal functions: subtest “inhibition capacity” of the Syndrom-Kurz-Test (SKT), phonetic verbal fluency, subtest “similarities” of the WAIS-III^39^, subtest inverse digits of the WAIS-III, clock test; (8) mood; and (9) Hospital Anxiety and Depression Scale (ADH).

Data from historical neuropsychological examinations performed at Ace Alzheimer Center Barcelona’s Memory Clinic within the 3 months prior to the participant’s inclusion were used for the current study.

#### Anthropometric measures

Additional assessments performed at baseline (v0), 12 (v2), and 24 months (v4) included vital signs and an anthropometric study carried out using a body composition monitor with bioimpedance technology (Tanita). Before the latter examination, participants had to remain at rest for 10 min. Data collected included heart rate, systolic pressure, diastolic pressure, weight, height, body mass index, percentage of fat mass, percentage of body water, percentage of muscle mass, and percentage of bone mass (Table 1).

### Ethical approval

The study protocol was approved by the Spanish Drug Agency (AEMPS for its initials in Spanish), with a protocol version 1 approval date of 15/03/2021, and was registered in the Spanish Clinical Trials registry (REEC) under the number 2020-003776-41 and the protocol version 2 extension was approved on 31/01/2022. The study was registered in ClinicalTrials.gov under the number NCT05594355 on 26/10/2022. The ethical committee of the Universitat Internacional de Catalunya reviewed and approved the protocol. The patient and a close relative or legal representative read the patient information sheet, agreed to participate in the trial, and then signed the informed consent form. The study was conducted in compliance with a clinical protocol, regulatory requirements, good clinical practice (GCP), and the ethical principles of the latest revision of the Declaration of Helsinki as adopted by the World Medical Association.

## Results

### Baseline characteristics of the study cohort

The inclusion of participants with MCI began in April 2021 and has reached the target of recruiting 100 participants by November 2021. Participants were randomized into two groups: one received EGb 761 (study group, *n* = 50), and the other did not receive any medication (control group, *n* = 50).

#### Sociodemographic characteristics

The baseline sociodemographic characteristics of the study sample are detailed in Table 2. The mean age of the participants was 73, with the majority being (60%) female and having completed a mean of 10 years of formal education. No significant differences in sociodemographic characteristics were detected between the control and study groups. A family history of cognitive impairment was present in 46% of participants.

**Table 2:** Baseline demographic, Tanita, and cardiovascular characteristics of the study participants.

Variable	Total study sample (*n* = 100)	Study group (*n* = 50)	Control group (*n* = 50)	*p* value
Age, mean (SD), years	73.1 (7.5)	71.9 (7.2)	74.2 (7.7)	0.126
Sex				0.414
Male, *n* (%)	40 (40.0)	18 (36.0)	22 (44.0)	
Female, *n* (%)	60 (60.0)	32 (64.0)	28 (56.0)	
Education, mean (SD), years	10.0 (4.2)	10.5 (4.2)	9.5 (4.3)	0.242
Years from MCI diagnosis, mean (SD)	3.1 (3.0)	2.8 (2.8)	3.3 (3.1)	0.399
Family history of cognitive impairment, *n* (%)	46 (46.0)	22 (44.0)	24 (48.0)	0.688
Body composition (Tanita) (SD)				
Fat mass (%)	35.2 (9.2)	35.6 (7.9)	34.9 (12.4)	0.737
Body water (%)	45.0 (5.4)	45.2 (3.8)	44.9 (11.0)	0.904
Muscular mass (%)	14.3 (3.5)	14.0 (3.6)	14.5 (4.5)	0.166
Bone mass (%)	3.3 (0.4)	3.3 (0.3)	3.2 (0.8)	0.411
Cardiovascular risk, mean (SD)				
Blood pressure, mmHg				
Systolic	136.9 (17.2)	134.9 (18.0)	138.9 (16.4)	0.248
Diastolic	79.8 (10.1)	79.4 (11.2)	80.1 (9.1)	0.732
Heart rate, bpm	66.7 (11.0)	66.4 (10.2)	66.9 (11.8)	0.821
Weight, kg	72.0 (13.2)	71.4 (11.7)	72.6 (17.8)	0.691
BMI, kg/m^2^	28.2 (4.9)	28.1 (3.7)	28.3 (7.1)	0.860
Diabetes, *n* (%)	16 (16.0)	4 (8.0)	12 (24.0)	0.054
Hypercholesterolemia, *n* (%)	56 (56.0)	32 (64.0)	24 (48.0)	0.107
Hypertension, *n* (%)	69 (69.0)	35 (70.0)	34 (68.0)	0.829
Smokers, *n* (%)	16 (16.0)	6 (12.0)	10 (20.0)	0.275

*SD* standard deviation.

#### Clinical characteristics

The mean time between the onset of cognitive symptoms and the diagnosis of MCI was 3.1 years. The most frequent cardiovascular risk factor was hypertension (69%), followed by hypercholesterolemia (56%), diabetes mellitus (16%), and smoking habits (16%). The mean BMI at baseline was 28 kg/m^2^, the mean systolic pressure was 137 mmHg, and the mean diastolic pressure was 80 mmHg. The body mass composition characteristics of participants are detailed in Table 2. Again, no significant differences in clinical characteristics were found between the control and study groups.

#### Cognitive characteristics

The baseline neuropsychological characteristics of the study participants are detailed in Table 3. The mean MMSE score was 26.7 ± 2.2, and the mean NPI-Q score was 2.5 ± 3.9. After the baseline assessment, the following syndromic cognitive diagnoses were found in the total study sample: possible amnestic MCI (37%) and possible non-amnestic MCI (63%). There were no significant differences between the groups in MMSE, CDR, Hachinski Ischemia Scale, NBACE scores, or amnestic profile. The total NPI-Q score did not differ significantly between the two groups, but the irritability/lability parameter showed higher scores in the control group (*p* = 0.006), as did the Blessed Dementia Scale (*p* = 0.048). However, after Bonferroni correction no significant differences were observed between the two groups.

**Table 3:** Baseline neuropsychological characteristics of the study participants.

Variable	Total study sample	Study group	Control group	*p* value
	(*n* = 100)	(*n* = 50)	(*n* = 50)	
Neurological evaluation, mean (SD)				
Mini-Mental State Examination (MMSE)	26.7 (2.2)	26.7 (2.4)	26.8 (2.0)	0.821
Clinical dementia rating (CDR)	0.5 (0.0)	0.5 (0.0)	0.5 (0.0)	–
Hachinski Ischemia Scale	2.0 (1.7)	1.9 (1.8)	2.1 (1.6)	0.558
Neuropsychiatric Inventory Questionnaire (NPI-Q)*	2.5 (4.0)	2.0 (3.8)	3.0 (4.1)	0.209
Symptoms, n (%)				
Delusions	0 (0.0)	0 (0.0)	0 (0.0)	–
Hallucinations	0 (0.0)	0 (0.0)	0 (0.0)	–
Agitation/aggression	2 (2.0)	1 (2.0)	1 (2.0)	1.000
Depression/dysphoria	41 (41.0)	20 (40.0)	21 (42.0)	0.839
Anxiety	38 (38.0)	16 (32.0)	22 (44.0)	0.216
Elation/euphoria	0 (0.0)	0 (0.0)	0 (0.0)	–
Apathy/indifference	18 (18.0)	6 (12.0)	12 (24.0)	0.118
Disinhibition	4 (4.0)	3 (6.0)	1 (2.0)	0.617
Irritability/lability	17 (17.0)	3 (6.0)	14 (28.0)	0.006
Motor disturbance	2 (2.0)	1 (2.0)	1 (2.0)	1.000
Nighttime behaviors	21 (21.0)	10 (20.0)	11 (22.0)	0.806
Appetite/eating	3 (30.0)	1 (2.0)	2 (4.0)	1.000
Geriatric Depression Scale	3.6 (2.6)	3.3 (2.3)	3.9 (2.8)	0.245
Blessed Scale (BDRS)	1.5 (1.3)	1.2 (1.2)	1.7 (1.3)	0.048
NBACE, mean (SD)				
Global orientation	14.6 (0.9)	14.6 (1.0)	14.7 (0.8)	0.618
Verbal learning WMS-III^#^	23.8 (6.6)	24.7 (6.5)	23.0 (6.6)	0.197
Delayed recall WMS-III	4.7 (2.9)	5.0 (2.9)	4.5 (3.0)	0.399
Recognition memory WMS-III	20.8 (3.0)	21.1 (3.0)	20.4 (3.0)	0.246
Digit span forward WAIS-III	7.1 (1.5)	7.1 (1.6)	7.1 (1.5)	1.000
Digit span backwards WAIS-III	4.3 (1.7)	4.1 (1.7)	4.5 (1.7)	0.242
Block design WAIS-III	3.0 (1.4)	2.8 (1.5)	3.2 (1.3)	0.157
Imitation praxis	3.3 (0.9)	3.3 (0.9)	3.3 (0.9)	1.000
Ideomotor praxis	4.0 (0.1)	4.0 (0.1)	4.0 (0.0)	1.000
Visual naming (15-BNT)	14.0 (1.5)	14.2 (1.1)	13.8 (1.8)	0.183
Poppelreuter’s test (responses)	9.5 (1.1)	9.4 (1.4)	9.5 (0.8)	0.662
15-OT (responses)	11.6 (2.5)	11.3 (2.9)	12.0 (2.5)	0.199
Luria’s clock test	3.3 (0.9)	3.3 (1.0)	3.3 (0.8)	1.000
Automatic inhibition SKT (s)	32.2 (10.7)	31.8 (13.7)	32.6 (12.2)	0.758
Automatic inhibition SKT (error)	1.9 (2.5)	2.2 (3.0)	1.6 (1.8)	0.229
Phonetic verbal fluency	12.0 (4.6)	12.1 (4.3)	11.9 (4.8)	0.827
Semantic verbal fluency	14.4 (5.5)	14.1 (5.1)	14.6 (6.0)	0.654
Similarities WAIS-III	10.1 (2.9)	10.0 (3.0)	10.2 (2.9)	0.735
Amnestic profile				
Possible amnestic MCI, *n* (%)	37 (37.0)	16 (32)	21 (42)	0.300

*SD* standard deviation, *WMS-III* Wechsler Memory Scale, Third Edition, *WAIS-III* Wechsler Adult Intelligence Scale, Third Edition, *15-BNT* the abbreviated Boston Naming Test with 15 items, *15-OT* The 15-Objects Test, *SKT* Syndrom-Kurz-Test, *s* time in seconds.

^#^Verbal learning WMS-III = 1st + 2nd + 3rd + 4th trial scores.

*Bonferroni-adjusted significance level of p value to 0.003 was calculated.

## Discussion

**Figure 2:**
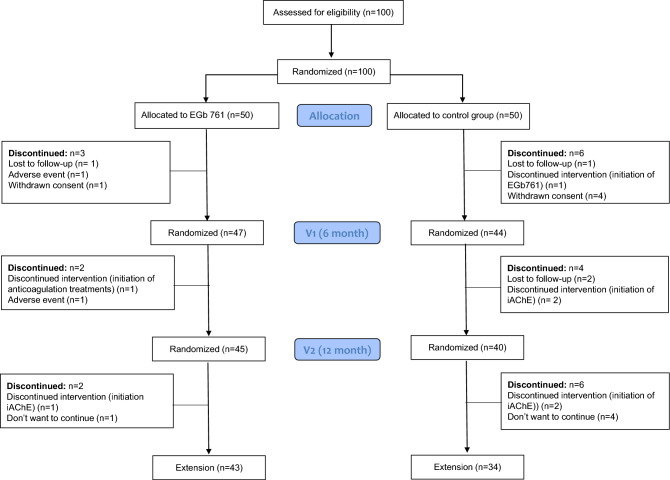
Flow of patients through the study.

In this study, we describe the procedures and baseline characteristics of an open-label, randomized, parallel-group controlled clinical trial on the long-term effect of EGb 761 treatment on inflammation and oxidative stress blood biomarkers, cognition, and neuropsychiatric symptoms in a cohort of 100 individuals with MCI recruited from a Memory Clinic and followed for 24 months. The study sample (*n* = 100) was enrolled in November 2021. All study participants have already completed the first 12 months of follow-up (v2) and are currently being followed in the extension phase (Fig. 2).

Importantly, the pathophysiology of MCI appears to be multifactorial. Across the spectrum of age-related cognitive impairment, neurodegenerative changes (i.e., neuronal loss and synaptic deficits) and histopathological alterations (i.e., increased production of β-amyloid leading to extracellular amyloid-containing plaques and the formation of intracellular hyperphosphorylated tau protein tangles) have been described^3^. Impaired cerebral glucose metabolism^37^, mitochondrial dysfunction, and increased oxidative stress have all been observed^8^. In addition to neurodegenerative brain changes, dementia is frequently associated with vascular pathologies such as small vessel disease (multiple or single infarcts)^38^.

EGb 761 extract has various beneficial properties, although its mechanism of action in cognitive disorders is not yet fully understood. Preclinical evidence suggests that EGb 761 acts on many of the previously described factors, including potent antioxidant activity, which reduces mitochondrial production of reactive oxygen species and Aβ-induced neuron toxicity by inhibiting the formation of Aβ oligomers^39–44^ and affects the insulin receptor by influencing acetylcholine reduction^41,42^. Its effects on neuronal function, neuroplasticity^43^, and neuroinflammation have also been described^44^. The available scientific evidence suggests that EGb 761 increases cerebral blood flow and brain perfusion by decreasing blood viscosity and protects blood vessels against processes involved in atherosclerosis^45,46^ without any relevant interactions of EGb 761 with the platelet anti-aggregatory effects of acetylsalicylic acid^47^. In addition, EGb 761 has also been shown to increase dopamine levels in the prefrontal cortex^48^.

Even though the mechanism of action of EGb 761 has been extensively studied using animal models, it is important to continue exploring its direct effects on humans to gain a comprehensive understanding of its effects on patients with cognitive impairment. With the quantification of two protein panels from Olink Proteomics over time, this study will deepen our knowledge about the mechanism of action of EGb 761 and the metabolic pathways affected, especially those related to inflammatory, oxidative stress, and degenerative processes. A better understanding of EGb 761’s effects on humans can contribute to the rational usage of the compound. According to current recommendations, it should only be used as a symptomatic agent. Our trial will analyze promising potential mechanisms of action as a modifier of the progression of cognitive impairment in the MCI stage.

Since its introduction to the market, EGb 761 has been widely used to improve cognitive deficits in many conditions, ranging from healthy aging to dementia. Many clinical trials have been published over the last few decades with positive outcomes in terms of symptomatic efficacy and safety, although the maximum duration of follow-up in these studies was limited to 12–52 weeks^49,50^.

However, it remains unclear whether EGb 761 has disease-modifying effects, delaying the progression from MCI to dementia. A large cohort study suggests that EGb 761 may help delay the progression from MCI to dementia, mainly in those patients who have received the drug for longer periods of time^51^. In a randomized, double-blind, 52-week trial, EGb 761 significantly reduced the progression from MCI to dementia and slowed cognitive decline^52^. On the other hand, in two randomized clinical trials, EGb 761 was unable to reduce the overall incidence rate of dementia or AD incidence in elderly individuals with normal cognition, subjective cognitive decline, or those with MCI. However, in both trials, dementia incidence was too low to draw definite conclusions regarding efficacy. In the US trial (NCT00010803)^53^, low treatment compliance might have also contributed to the negative outcome^54^. In addition, the French study conducted by Vellas et al.^55^ was inconclusive due to the fact that the pre-specified statistical test assumed proportional hazards, which was not the case. A post hoc analysis demonstrated a significantly lower rate of progression to AD in the EGb 761 group using an appropriate statistical test^55^.

Currently, there are no FDA-approved medications for the treatment of MCI; therefore, longitudinal and high-quality studies are required to identify pharmacologic and/or dietary agents that might improve cognition or delay progression in patients with MCI. Although different drug agencies have approved cholinesterase inhibitors (AChEIs) for the treatment of Alzheimer’s disease, their benefits for the treatment of MCI remain inconclusive^56^. Multiple guidelines currently recommend EGb 761 for the symptomatic treatment of MCI^57^, however, the specific mechanism of action underlying these effects has never been investigated in plasma. There is also evidence that EGb 761 may help delay the progression from MCI to dementia in some individuals, mainly those who have received the drug for longer periods^58^. Furthermore, the effects of EGb 761 on cerebrovascular blood flow may also contribute to the clinical benefits observed in MCI patients with underlying cerebrovascular disease^59^.

In the present clinical trial, we also aim to assess the clinical impact of EGb 761 on cognitive functions and behavioral symptoms in the long term (24-month follow-up) in patients with MCI. This study will provide data beyond the 52 weeks available in the currently published studies^13^, demonstrating the effect of continuing EGb 761 treatment for longer periods. The fact that our study has a control group (participants who did not receive EGb 761 treatment for the first 12 months) will allow us to check the natural progression of inflammatory and oxidative blood biomarkers and changes in cognitive impairment in MCI for the first year of the study and in the extension phase (in which treatment with EGb 761 will be initiated in participants in the control group). The information the study will provide once treatment with EGb 761 is discontinued is also important. These data will aid in assessing the effect of treatment discontinuation on cognitive functions and biomarkers.

We acknowledge that our study has several limitations. First, we lack information from AD core biomarkers regarding the underlying etiology of MCI AT(N) profiles (amyloid beta [A], pathologic tau [T], and neurodegeneration [N])^60^. Plasma AD biomarkers will also be assessed to characterize all study participants. Second, we lack neuroimaging to assess the cerebrovascular burden in our participants. Third, the study sample size is relatively small, but based on sample size calculations, it should be sufficient to evaluate significant changes in inflammatory and oxidative biomarkers. Fourth, the primary outcome measures of our study, neuroinflammation and oxidative biomarkers, will be measured in the periphery (blood) and not in CSF. Lastly, we recognize that knowledge of treatment assignment may affect patient-reported outcomes (PROs).

A better understanding of the mechanisms underlying the neuroprotective effects and potentially disease-modifying mechanisms of EGb 761 may contribute to a better understanding of the effectiveness and complexity of this drug and may also aid in the design of future clinical therapeutic strategies.

## Data Availability

The datasets generated and/or analyzed for this study will be made available by the corresponding author upon reasonable request.
